# Performances of the Canadian Agility and Movement Skill Assessment (CAMSA), and validity of timing components in comparison with three commonly used agility tests in Chinese boys: an exploratory study

**DOI:** 10.7717/peerj.8784

**Published:** 2020-03-23

**Authors:** Yue Cao, Chunhua Zhang, Rong Guo, Dandan Zhang, Shijiao Wang

**Affiliations:** School of Kinesiology, Shanghai University of Sport, Shanghai, China

**Keywords:** Children, Fundamental movement skills, Agility capacity, Validity, Testing

## Abstract

**Background:**

The practical application of the Canadian Agility and Movement Skill Assessment (CAMSA) has been reported in some Western countries. However, a few studies reported the application of the CAMSA in Chinese children. In addition, given that the CAMSA was designing to incorporate both movement skills and agility assessment, the value and validity of the timing component of the CAMSA are worth discussing.

**Methods:**

By choosing the Illinois Agility Test, Repeated Side Step-1 m distance, and the newly designed Repeated Side Step-half of height as the benchmark, we evaluate the performance of the CAMSA, further establish the concurrent validity of the CAMSA timing components (completion time and time score). In total, 149 male children (mean age 9.0 ± 0.8 years) from public schools in Shanghai, China, participated in the study.

**Results:**

The mean CAMSA completion time was 19.3 ± 5.3 (s), and mean time score was 8.7 ± 3.9 (range of 1–14) for all participants (*n* = 149). After adjusted the sprint speed, older age was positively associated with the performance of the CAMSA. Being overweight was not associated with the performance of the CAMSA comparing with healthy body mass children, however, being obese was negatively associated with the CAMSA timing components and total score. Children having extracurricular sports activities (e.g., athletic experiences), mostly soccer, were more likely to demonstrated better performances of the CAMSA completion time, time score and total score. However, overweight and obese, also athletic experiences were not significantly contributed to the CAMSA skill score, although the association was slight (Adj *R*^2^ = 0.13). Besides, the CAMSA completion time has a strong correlation with the IAT, *r* = 0.77; RSS-1MD, *r* =  − 0.76; and RSS-HHD, *r* =  − 0.77, *p* < 0.01. The same pattern of correlation was also found between the CAMSA time score and three agility tests: IAT, *r* =  − 0.79; RSS-1MD, *r* = 0.76; RSS-HHD, *r* = 0.78, *p* < 0.01.

**Discussion:**

Overall, a few participants in the study were able to reach the recommended level of the total CAMSA score referring to the Canadian criterion. The strong concurrent validity was found between the CAMSA timing components and three selected agility tests, respectively.

## Introduction

There are three main categories of fundamental movement skills (FMS): object control, locomotor skills, and stability skills (Gallahue, Ozmun & Goodway, 2012). Recent research, though, suggests expanding the concept range of “traditional” FMS (Hulteen et al., 2018), it is noted from longitudinal studies and large-scale surveillances that motor competence is associated with overweight/obese and other health-related physical fitness (Barnett et al., 2008; D’Hondt et al., 2012; Hu, Wang & Li, 2018; Lubans et al., 2010), also participation in physical activity (Han et al., 2018; Holfelder & Schott, 2014) throughout the lifespan (Hulteen et al., 2018). To date, many FMS assessments have been applied or revised to observe the development and/or mastery of movement skills in children worldwide (Brown & Lalor, 2009; Duger et al., 1999; Logan et al., 2017; Williams et al., 2009). Indeed, low movement skill proficiency (e.g., non-mastery of object-control and locomotor skills) is prevalent among children, especially in elementary school-age students (Hardy et al., 2012). In recent years, China has been devoting a lot of attention to physical health, movement skills, and specialized sports training of children through different policies (Zhang & Yang, 2017). Although few object control skill assessments (e.g., soccer, basketball, volleyball) and gymnastic skills have become part of the junior high school entrance examinations in certain areas of China (e.g., Beijing, Shanghai) (BMEC, 2018; Shanghai Municipal Education Committee, 2019), the assessment with comprehensive aspects of FMS is still lacking in the latest version of the national surveillance (e.g., the Chinese National Student Physical Fitness Standard (revised version, 2014)) (Ministry of Education of the People’s Republic of China, 2014). There is clearly a need for a feasible FMS assessment that could be conducted on school campuses for large-scale population testing.

The Canadian Agility and Movement Skill Assessment (CAMSA) (Longmuir et al., 2017) has been recently developed and promoted nationwide in Canada as one of the components of the Canadian Assessment of Physical Literacy (CAPL) (Francis et al., 2016). It was known as the “obstacle course” in the 2014 CAPL-1 (Longmuir et al., 2015), and, once evaluation norms were established for all children aged 8–12 years, it was revised in the 2017 CAPL-2. The CAMSA requires the participant to perform a combined action of seven movement skills in a set-up that simulates the dynamic situation of daily physical activity and training as quickly as possible. The performance of each skill and the overall completion time are scored individually, and the individual scores are then combined to a total score. A better CAMSA total score indicates that participants can demonstrate not only each fundamental movement skill (e.g., catch a ball, use an overhand throw to hit a target, kick a soccer ball); but also the combined and complex movement skills more accurately (e.g., sliding sideways with low centre of gravity and touching the cones, kick a soccer ball with continuous running pattern). A better total score might also indicates that participants are having better agility capacity (Sheppard & Young, 2006) to shift quickly from different body positions (e.g., S-type one-foot hopping six times), and different moving directions (e.g., from sagittal two-foot jumping three times into sliding sideways), also in quick response to a stimulus (e.g., catch a ball thrown by one examiner).

Recent studies have reported a moderate to excellent retest reliability of the CAMSA over short and long intervals (Longmuir et al., 2017). The feasibility of the CAMSA was confirmed via a shorter testing time (5–7 min set-up time and 1 min testing time) and flexible environmental requirements (both indoor and outdoor testing are available) (Longmuir et al., 2017), the application within a school Physical Education (PE) class (Lander et al., 2015), and also the small effect of an examiner’s professional training background on the CAMSA performances (Law et al., 2018). Although many studies have indicated that the CAMSA could be a feasible assessment, study participants were usually recruited from western countries and rarely from Asia. While the purpose of the CAMSA is to incorporate the assessment of movement skills and the agility capacity, a strong concurrent validity was also found of the CAMSA skill components (e.g., skill score) in comparison with a redesigned assessment protocol, which was based on an existing FMS assessment (Lander et al., 2017). However, the correlation between the CAMSA timing components (e.g., completion time and time score) and traditional agility tests remains unclear.

In summary, the main purpose of this study was to evaluated the performance of the CAMSA as well as established the concurrent validity of the CAMSA timing components in comparison with three agility benchmarks: the Illinois Agility Test (IAT), Repeated Side Step-1 m distance (RSS-1MD) and Repeated Side Step-half of height (RSS-HHD), among male elementary school children in China.

## Materials & Methods

### Study design and participants

The definition of agility is considered as a rapid whole-body movement with change of velocity or direction in response to a stimulus (Sheppard & Young, 2006). Agility tests (Sheppard & Young, 2006; Stewart, Turner & Miller, 2014) often involve sprint and change-of-direction movements toward a specifically route of motion (e.g., side-stepping, running or dribbling through the obstacle) (Kutlu et al., 2012; Lockie et al., 2013). Unfortunately, it is difficult to find one existing agility test that was able to represent the comprehensive idea of agility as the perfect benchmark (e.g., “gold standard”).

In order to established the validity evidence of the CAMSA completion time and time score, among commonly used tests, this study chose three agility tests as the benchmark: the IAT and two versions of RSS following three reasons: (1) both the IAT and CAMSA require participants to complete the assessment as quickly as possible, using total completion time as the outcome; (2) The IAT also includes change-of-direction movements (e.g., lying prone shift to running and change of direction, shuttle running, swerving across cones). The retest reliability and concurrent validity of the IAT have shown to be excellent in children and adults (Hachana et al., 2013; Hachana et al., 2014; Raya et al., 2013); and (3) The RSS-1MD has commonly been used in Japan as an agility test for children (Morita et al., 2015), and includes change-of-direction movements in a short distance, which is in the CAMSA. (4) Considering the biomechanical factors of the hip, knee, and ankle joints changed at different side-step distances based on participant’s height in the RSS (Inaba et al., 2012) as well as the feasibility of testing, both the original RSS-1MD (e.g., the step distance is 1 m) and the new version designed by research team: RSS-HHD (e.g., the step distance is equal to the half of each participant’s height) were conducted in this study.

A convenience sample of male students (*n* = 149) was recruited from public elementary schools in Shanghai, China, to participate in this study. Written consent from the parents and verbal assent from the children was obtained for all participants prior to participation. Individuals were excluded if they were under eight years old, based on the applicable-age of the CAMSA criterion, or if they have had an injury or surgery in the past three months that restricted them from physical exercise. All participants should attend school PE classes (instructed sports and recreational activities, 45 min per-lesson), and were allowed to participate in extracurricular sports activities: school sport team (non-professional, usually train three times a week after school) and other community-based courses (teaching a specific sport e.g., soccer, football, usually train at the weekend). The study was approved by the Ethics Committee of Shanghai University of Sport in 2019 (No: 102772019RT049).

### Testing procedures

Tests were conducted in groups, which simulated the organization of PE classes, in the morning of a school day by four examiners, each with a graduate degree in kinesiology. One was identified as the lead examiner. All examiners had prior training in administering the CAMSA and used the official manual of the CAPL-2 edition. First, participants were asked to self-report their age and athletic experiences in the last month (e.g., extracurricular sports activities). Children were then coded and divided randomly into different groups, with about 20 participants in each group.

The anthropometric measurements (height and body mass), 50 m sprint, the IAT, RSS-1MD, RSS-HHD, and CAMSA were selected as indicators in this study. In order to fit in with the school’s daily schedules, testing procedures generally contained two days for each group. The anthropometrics were measured on the first day, followed by no more than two of three of the IAT, RSS (two versions at a time), or CAMSA, the remaining assessments were measured on the next day under the same condition. The whole group of participants should complete one assessment before going to the next. The 50 m sprint was conducted on school PE classes by teachers on other days in the same week when there were no study assessments.

Participants performed two timed trials of the IAT, RSS-1MD, RSS-HHD, and CAMSA in an outdoor soccer field after they had completed two practice trials with no mistakes. A twenty-minute rest period was given as participants finished one practice or timed trial and went back to wait at the end of the group line. Verbal corrections were provided during the practice trials. Only cues, such as the order of the CAMSA skills and the route of IAT, were provided during the timed trials, and no encouraging cues were provided.

The timed trial 2 of the CAMSA was selected as the outcome. The best performance between timed trials 1 and 2 of IAT the (shorter total completion time), RSS-1MD and RSS-HHD (more total accurate steps) were selected as the final outcome.

### Anthropometrics

Following the standardized protocol of Chinese National Student Physical Fitness Standard (revised version, 2014)(Ministry of Education of the People’s Republic of China, 2014), height (to the nearest 0.1 cm), body mass (to the nearest 0.1 kg) were measured using a portable instrument (GMCS-IV; Jianmin, Beijing, China) in bare feet and light clothes to calculate participant’s body mass index (BMI). The BMI criterion of overweight and obese defined by the Chinese National Student Physical Fitness Standard (revised version, 2014): for boys, age 8 (overweight: 19.5–22.1, obese: ≥22.2); age 9 (overweight: 20.2–22.6, obese: ≥22.7); age 10 (overweight: 21.5–24.1, obese: ≥24.2).

### 50 m sprint

Sprint speed is considered one of the import characteristics for young athletes (Negra et al., 2017), especially for soccer athletes (Reilly et al., 2000). The 50 m sprint was selected as one adjustment variable as the recruited participant could have athletic experiences of sports. To address the sprint speed in this study, participants were instructed to run in a straight line for 50 m on a flat and clear surface as fast as possible. Following the standardized protocol of Chinese National Student Physical Fitness Standard (revised version, 2014) (Zhu et al., 2017), test was performed once as a single maximum sprint for each participant. Two participants were tested at a time. The outcome for the 50 m sprint was recorded to the nearest 0.1s.

### Illinois agility test

**Figure 1:**
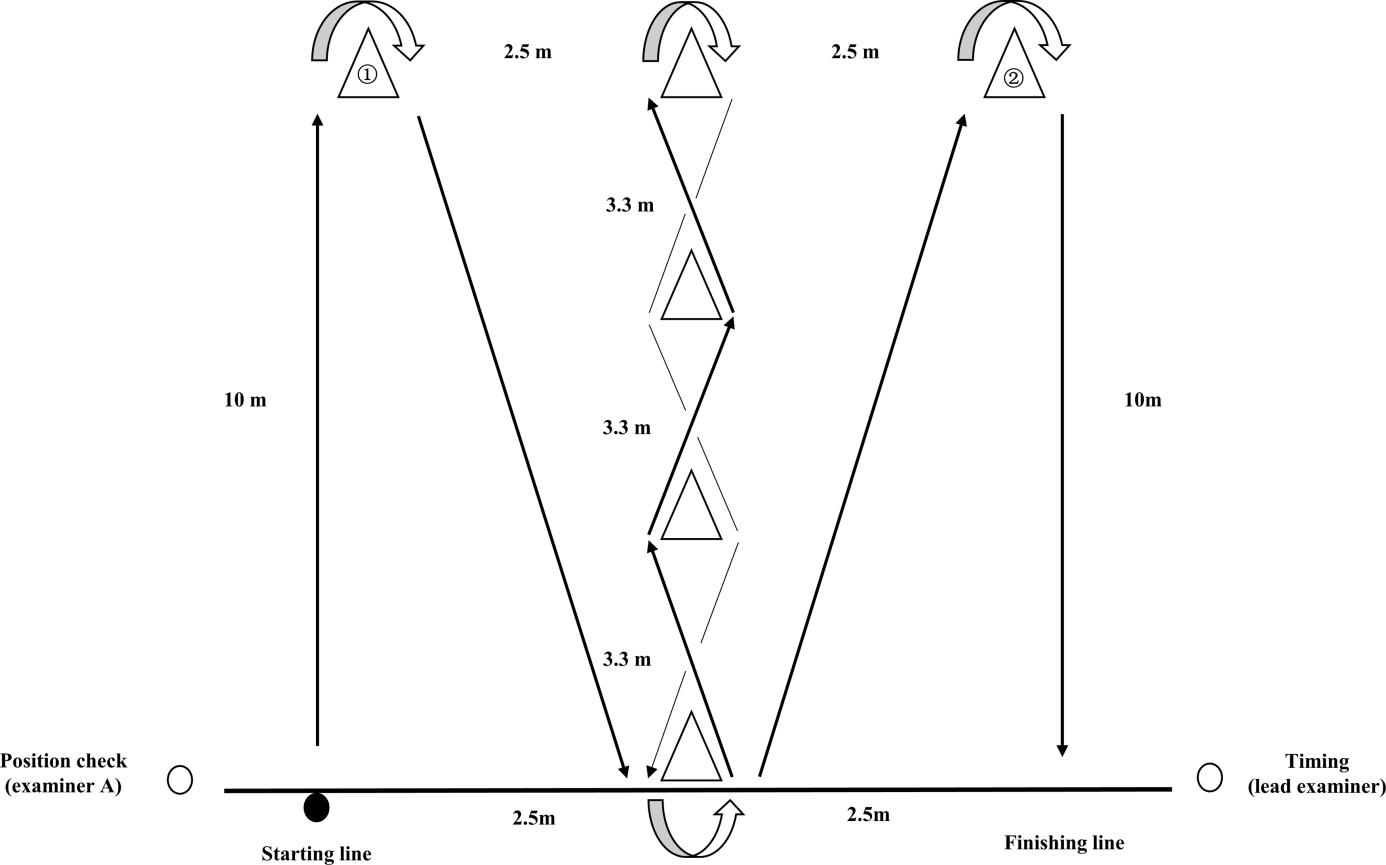
Illinois agility test layout. The participant started at the starting line and worked their way through the cones following the arrow until running across the finish line.

The layout of IAT was set as follows: the distance for the sagittal sprint (and return) was 10 m, the center cones spaced 3.3 m from each other for changing direction in a short distance (see Fig. 1). Testing started in a ready position with the participant lying prone on the floor with hands placed to the side at shoulder level and head behind the starting line. On the “go” command, the participant immediately changed position sprinting to cone 1, returned, swerved across four cones eight times (four in the reverse direction), ran across cone 2, and then headed back to the finish line. The outcome was measured as total completion time (to the nearest 0.1 s) from the start of position changing to when the participant ran across the finish line. The timing of the IAT was conducted by the lead examiner in the field, one assistant examiner was responsible for checking the ready position. Participants were instructed to complete the IAT following a fixed route without knocking down any cones. If the participant did knock a cone, the attempt was voided and the participant should start from the beginning.

### Repeated side step

**Figure 2:**
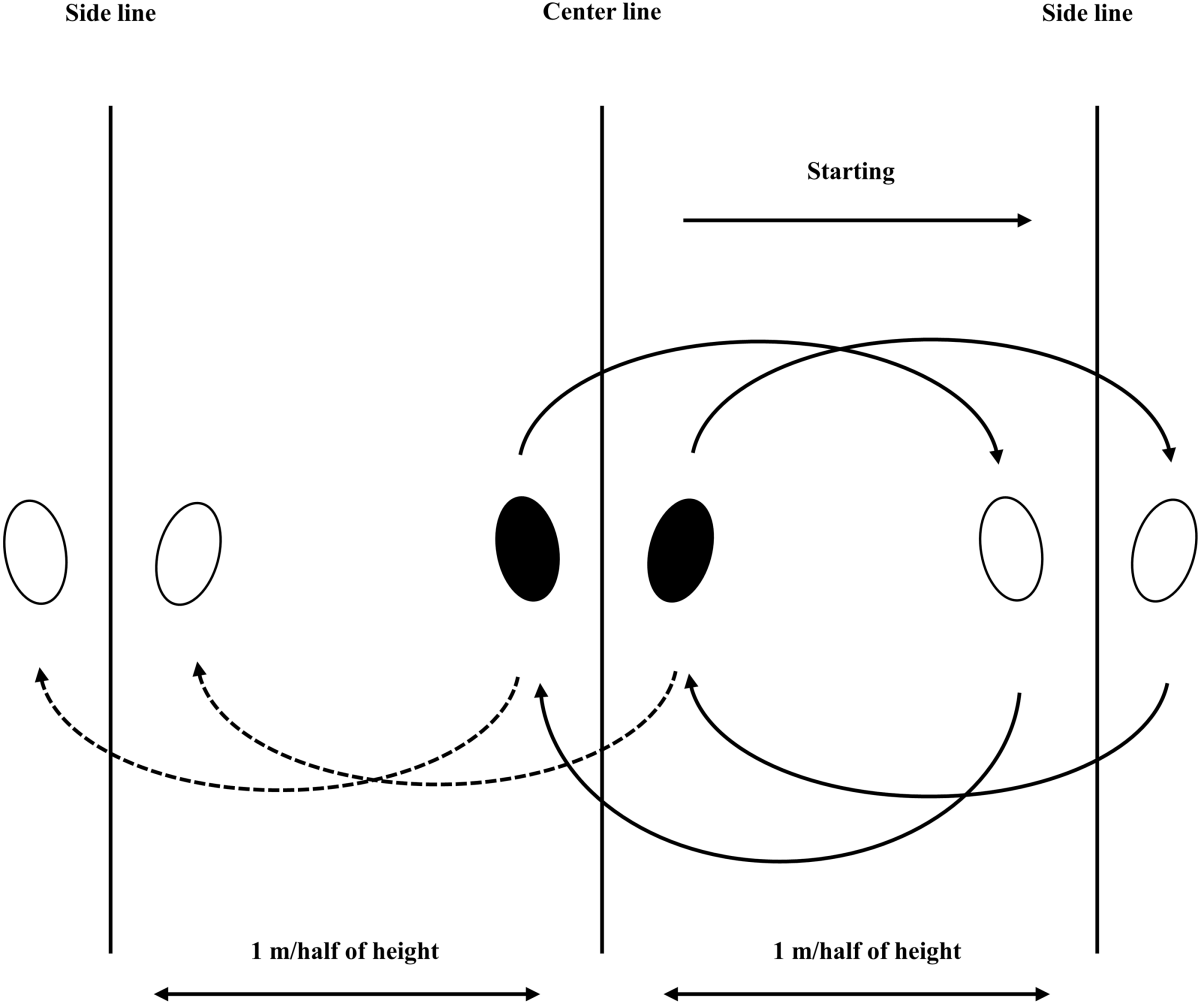
Repeated side step layout. The participant started at the center line and stepped to their right until their right foot crossed the right side line, then stepped back to center line, and repeated in left, center, and fight line in 20 s.

The RSS test requires participants to demonstrate as many as possible repeated sideward steps in 20 s. Two versions of the step distance (e.g., from the center line to each side line) were set (see Fig. 2). Participants demonstrated RSS-1MD first, then RSS-HHD. Testing started in a ready position with feet placed on both sides of the center line, facing forward throughout the trial. On the “go” command, the participant took one sideward step to the right (e.g., pushed off the floor with their right foot, left foot brought to meet and land with right foot) until their right foot had crossed the right side line. The participant then stepped back the center line and stepped in the order of left, center, right, and center. The outcome was measured as total accurate steps. Participants were instructed to perform the accurate pattern of one foot pushing off, if participants performed one step with both left and right foot pushed off at the same time, this step was considered as side-hops and was not counted in the total accurate steps. The step was also not counted in the total accurate steps when participants stepped on any lines or did not land with feet placed on both sides of the line. The inaccurate steps were warned during testing, however, testing would not be suspended due to inaccurate steps.

### Canadian agility and movement skill assessment

**Figure 3:**
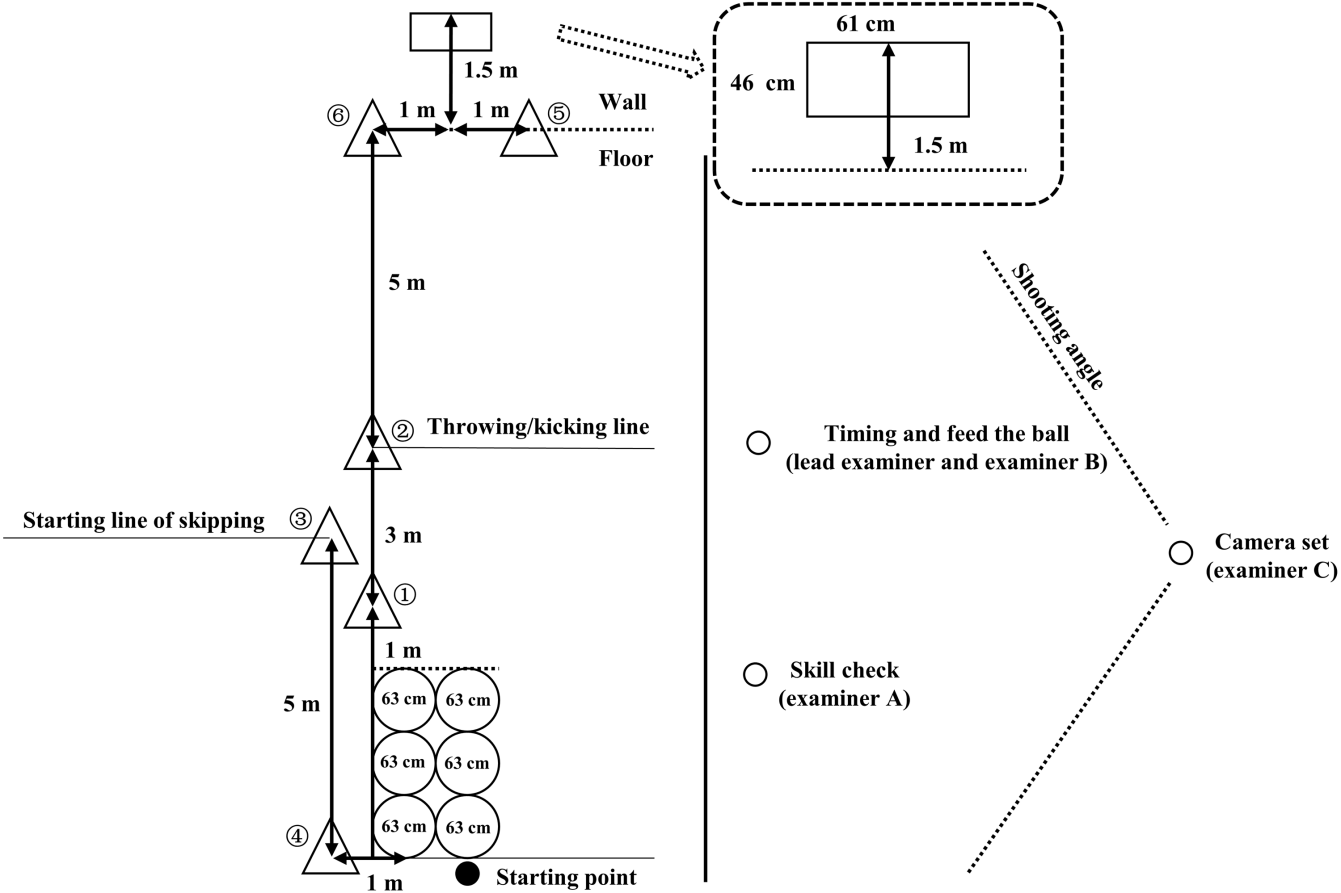
Canadian agility and movement skill assessment layout (adapted from the CAPL-2 edition). The participant started at the hoops and worked their way through the cones in numbered order before going through the hoops a second time.

The CAMSA requires participants to demonstrate dynamic movement skills in an open space (see Fig. 3). The CAMSA consisted of four components: completion time, time score, skill score, and total score. The time score was converted from completion time into a 14-point score (range of 1–14), with a higher score representing a shorter completion time on the CAMSA. The skill score was composed of 14 criterion points in seven movement skills (range of 0–14), with a score of 1 awarded for completing each skill criterion point or a score of 0 when one skill criterion point was not completed (e.g., “hops only once in each hoop without touching hoops”, awarded 1 point; or “touching of hoops for any reason”, awarded 0 point). Time and skill scores were weighted equally in the total CAMSA score (range of 1–28). During testing, the participant was instructed to stand at the starting point until a “go” command was given by the lead examiner. Then, timing started and the participant began two-foot jumping (past three hoops), followed by sliding (two times, one in the reverse direction), catching (reacted to the ball thrown by one examiner), throwing (at the target), skipping (for 5 m), one-foot hopping (past six hoops) and kicking (kick between two cones). The timing was stopped when the participant kicked the soccer ball, and total completion time was recorded (to the nearest 0.1 s).

During testing, timed trials of the CAMSA were video recorded and analysed after testing to estimate skill score. The timing of the CAMSA was conducted by the lead examiner in the field. One assistant examiner operated the camera, another threw a soft ball to the participant and placed the soccer ball at the kicking line in the CAMSA, the remaining examiner was responsible for marking incorrect skill performances of the CAMSA (e.g., missed the target in throwing or touched any hoops in hopping), which might not be clearly captured by the camera due to the shooting angle.

The skill score was given from the lead examiner. In addition, we asked three assistant examiners to give skill score independently for each participant to establish the inter-rater reliability of the CAMSA skill score (four examiners total).

### Statistical analysis

Statistical analysis was performed using SPSS 20.0. The significance level was set at *p* < 0.05. For descriptive analyses, results were presented as mean ± SD for anthropometrics, 50 m sprint, the IAT, two versions of RSS, and CAMSA. Scores of seven isolated CAMSA skills were presented as the median (Q25, Q75). To address the main aims of the study, a series of multiple linear regression analyses were conducted to examine whether demographic characteristics, body composition were associated with better performances of the assessment. Pearson’s correlation analysis was performed to establish the concurrent validity between the CAMSA components and the IAT, RSS-1MD, and RSS-HHD, respectively. Paired *t*-tests or Wilcoxon signed rank tests were used to examine the difference between two timed trials of the assessment. The inter-class correlation coefficient (ICC) was calculated to determine the inter-rater reliability for the CAMSA skill scores. The effect of (*r*) was considered: <0.3 as low, 0.30 to 0.49 as moderate, 0.5 and above as strong (Cohen, 1988). The strength of ICC was considered: 0.41 to 0.60 as fair, 0.61 to 0.80 as substantial, 0.81 and above as excellent (Landis & Koch, 1977).

## Results

Overall, 149 participants (mean age 9.0 ± 0.8 years) completed all assessments. Study participants were 142.9 ± 8.9 cm in height and 40.7 ± 11.9 kg in body mass. Body mass index was 19.6 ± 3.9 kg/m^2^, and 24 (16.1%) of participants were overweight, 31 (20.8%) were obese. The 50 m sprint was 10.4 ± 1.4 s for all participants. Just over one third (46, 30.9%) were involved in extracurricular sports activities, 39 of them were school soccer team members; 7 participants have attended community-based courses (e.g., soccer and football).

Table 1 displays descriptive information about two timed trials and the final outcome of the IAT, two versions of RSS, and CAMSA. The total completion time of the IAT on the first timed trial was statistically better than a second (*p* < 0.001, 95% CI [ −0.56, −0.17]). The total accurate steps of the RSS-1MD (*p* = 0.012, 95% CI [ −1.06, −0.13]) and RSS-HHD (*p* < 0.001, 95% CI [ −1.88, −0.62]) improved significantly on the second timed trial. Participants performed better CAMSA completion time (*p* < 0.001, 95% CI [0.83, 1.53]), time score (*p* < 0.001, 95% CI [ −1.18, −0.70]), and total score (*p* < 0.001, 95% CI [ −1.25, −0.56]) on the second timed trial, however, there was no difference in skill score (*p* = 0.78, 95% CI [ −0.21, 0.27]) between timed trials 1 and 2. There were no significant differences in most of the CAMSA skills between two timed trials, except the sliding skill (*p* = 0.013, *Z* =  − 2.48).

**Table 1:** The performance of the IAT, RSS-1MD, RSS-HHD, and CAMSA for all study participants (*n* = 149) between two timed trials.

**Variables**	**Timed trial 1**	**Timed trial 2**	Final outcome*
**IAT (s)**	23.5 ± 3.1	23.8 ± 3.2	23.2 ± 2.9
**RSS-1MD (steps)**	29.4 ± 5.7	30.0 ± 5.4	30.9 ± 5.3
**RSS-HHD (steps)**	33.0 ± 7.6	34.2 ± 7.4	35.1 ± 7.5
**CAMSA completion time (s)**	20.5 ± 5.6	19.3 ± 5.3	19.3 ± 5.3
**CAMSA time score (1–14)**	7.8 ± 3.9 (1–14)	8.7 ± 3.9 (1–14)	–
**CAMSA skill score (0–14)**	11.1 ± 1.9 (4–14)	11.1 ± 1.8 (6–14)	–
*Two-foot jumping* (0–2)	2 (2, 2)	2 (2, 2)	–
*Sliding* (0–3)	3 (3, 3)	3 (2, 3 )	–
*Catching* (0–1)	1 (1, 1)	1 (1, 1)	–
*Throwing* (0–2)	1 (1, 1)	1 (1, 1)	–
*Skipping* (0–2)	2 (2, 2)	2 (2, 2)	–
*One-foot hopping* (0–2)	1 (0.5, 2)	1 (0, 2)	–
*Kicking* (0–2)	2 (2, 2)	2 (2, 2)	–
**Total CAMSA score (1–28)**	18.9 ± 5.1 (6–27)	19.8 ± 5.0 (7–28)	–

**Notes.**

*The timed trial 2 of the CAMSA was selected as the final outcome. *The best performance between timed trials 1 and 2 of IAT the (shorter total completion time), RSS-1MD and RSS-HHD (more total accurate steps) were selected as the final outcome. Values are presented as mean ± SD or median (Q25, Q75). Score range was presented for CAMSA time, skill, and total score.

IAT Illinois Agility Test RSS-1MD Repeated Side Step1 m distance RSS-HHD Repeated Side Stephalf of height distance CAMSA Canadian Agility and Movement Skill Assessment

Participants performed poor in throwing skill with a median score (Q25, Q75) of 1 (1, 1), also in one-foot hopping with a score of 1 (0, 2). The full marks are 2 for throwing and one-foot hopping. Although the skill score of the CAMSA was given from the lead examiner, the inter-rater reliability (four examiners assisted in this study) of skill scores were substantial for timed trial 1 (ICC = 0.71, 95% CI [0.59, 0.79]) and timed trial 2 (ICC = 0.62, 95% CI [0.48, 0.72]).

**Table 2:** Multiple linear regression examining the influence of age, training experiences, body composition on whether participants achieve better performances on the IAT, RSS-1MD, RSS-HHD, and CAMSA (*n* = 149).

**Models**	**Adj *R*^2^**	**B**	**SE**	**Beta**	***t***	**Sig**	**95% CI**
**IAT (s)**	0.73						
*Age*		−0.20	0.15	−0.06	−1.33	0.186	(−0.50, 0.10)
*Overweight*		1.00	0.36	0.13	2.76	0.007	(0.28, 1.71)
*Obese*		1.69	0.35	0.24	4.81	<0.001	(1.00, 2.39)
*Having athletic experiences*		−1.65	0.35	−0.26	−4.72	<0.001	(−2.34, −0.96)
*50 m sprint*		1.12	0.12	0.52	9.07	<0.001	(0.88, 1.37)
**RSS-1MD (steps)**	0.56						
*Age*		0.65	0.35	0.10	1.83	0.069	(−0.05, 1.34)
*Overweight*		0.24	0.85	0.02	0.28	0.783	(−1.45, 1.92)
*Obese*		−1.64	0.83	−0.12	−1.97	0.050	(−3.28, 0.00)
*Having athletic experiences*		4.02	0.82	0.35	4.87	<0.001	(2.39, 5.65)
*50 m sprint*		−1.66	0.29	−0.42	−5.69	<0.001	(−2.24, −1.08)
**RSS-HHD (steps)**	0.58						
*Age*		1.96	0.48	0.22	4.06	<0.001	(1.00, 2.92)
*Overweight*		−0.58	1.17	−0.03	−0.50	0.618	(−2.89, 1.72)
*Obese*		−3.34	1.14	−0.18	−2.94	0.004	(−5.58, −1.09)
*Having athletic experiences*		5.50	1.13	0.34	4.87	<0.001	(3.27, 7.74)
*50 m sprint*		−2.09	0.40	−0.38	−5.22	<0.001	(−2.88, −1.30)
**CAMSA completion time (s)**	0.56						
*Age*		−1.77	0.35	−0.28	−5.05	<0.001	(−2.46, −1.08)
*Overweight*		1.29	0.85	0.09	1.52	0.131	(−0.39, 2.96)
*Obese*		3.34	0.82	0.26	4.05	<0.001	(1.71, 4.97)
*Having athletic experiences*		−3.31	0.82	−0.29	−4.04	<0.001	(−4.93, −1.69)
*50 m sprint*		1.31	0.29	0.33	4.52	<0.001	(0.74, 1.88)
**CAMSA time score**	0.62						
*Age*		1.29	0.24	0.28	5.41	<0.001	(0.82, 1.76)
*Overweight*		−0.84	0.58	−0.08	−1.46	0.145	(−1.98, 0.30)
*Obese*		−1.98	0.56	−0.21	−3.52	0.001	(−3.08, −0.87)
*Having athletic experiences*		3.07	0.56	0.37	5.50	<0.001	(1.97, 4.17)
*50 m sprint*		−0.96	0.20	−0.34	−4.88	<0.001	(−1.35, −0.57)
**CAMSA skill score**	0.13						
*Age*		0.33	0.16	0.16	2.04	0.044	(0.01, 0.66)
*Overweight*		0.27	0.40	0.06	0.69	0.488	(−0.51, 1.06)
*Obese*		−0.46	0.38	−0.11	−1.19	0.237	(−1.22, 0.30)
*Having athletic experiences*		0.06	0.38	0.02	0.17	0.867	(−0.69, 0.82)
*50 m sprint*		−0.39	0.14	−0.30	−2.89	0.004	(−0.66, −0.12)
**Total CAMSA score**	0.56						
*Age*		1.62	0.33	0.28	4.94	<0.001	(0.97, 2.27)
*Overweight*		−0.57	0.79	−0.04	−0.72	0.474	(−2.14, 1.00)
*Obese*		−2.43	0.77	−0.20	−3.15	0.002	(−3.96, −0.90)
*Having athletic experiences*		3.13	0.77	0.29	4.08	<0.001	(1.61, 4.65)
*50 m sprint*		−1.35	0.27	−0.37	−4.98	<0.001	(−1.89, −0.82)

**Notes.**

For all models, the reference categories of overweight and obese were healthy body mass participants, respectively. The BMI criteria of overweight and obese defined by the Chinese National Student Physical Fitness Standard (revised version, 2014).

IAT model: *F* = 82.16, *p* < 0.001. RSS-1MD model: *F* = 38.43, *p* < 0.001. RSS-HHD model: *F* = 41.71, *p* < 0.001. CAMSA completion time model: *F* = 38.77, *p* < 0.001. CAMSA time score model: *F* = 49.00, *p* < 0.001. CAMSA skill score model: *F* = 5.49, *p* < 0.001. Total CAMSA score model: *F* = 37.87, *p* < 0.001.

IAT Illinois Agility Test RSS-1MD Repeated Side Step1 m distance RSS-HHD Repeated Side Stephalf of height distance CAMSA Canadian Agility and Movement Skill Assessment

Linear regression results are presented in Table 2. After adjusted sprinting, participants with athletic experiences were more likely to demonstrate better performances in agility tests: the IAT (Adj *R*^2^ = 0.73, *p* < 0.001, 95% CI [ −2.34, −0.96]), RSS-1MD (Adj *R*^2^ = 0.56, *p* < 0.001, 95% CI [2.39, 5.65]), and RSS-HHD (Adj *R*^2^ = 0.58, *p* = 0.001, 95% CI [3.27, 7.74]), also CAMSA completion time (Adj *R*^2^ = 0.56, *p* < 0.001, 95% CI [ −4.93, −1.69]), time score (Adj *R*^2^ = 0.62, *p* < 0.001, 95% CI [1.97, 4.17]), total score (Adj *R*^2^ = 0.56, *p* < 0.001, 95% CI [1.61, 4.65]). Having athletic experiences did not statistically influence the CAMSA skill score (*p* = 0.867, 95% CI [ −0.69, 0.82]), although the association was slight (Adj *R*^2^ = 0.13). Older children were getting better in RSS-HHD (*p* < 0.001, 95% CI [1.00, 2.92]), same pattern of positive associations were observed in the CAMSA (*p* < 0.05). Being overweight and obese were more likely to demonstrate a worse result of IAT (*p* < 0.05). Besides, obese participants were more likely to demonstrate less total accurate steps of the RSS-HHD (*p* = 0.004, 95% CI [ −5.58, −1.09]). Being obese was negatively associated with the CAMSA completion time (*p* < 0.001, 95% CI [1.71, 4.97]), time score (*p* = 0.001, 95% CI [ −3.08, −0.87]), and total score (*p* = 0.002, 95% CI [ −3.96, −0.90]), while being overweight did not statistically contribute to RSS-1MD, RSS-HHD, CAMSA timing components and total score (*p* > 0.05). Moreover, being overweight and obese did not statistically contribute to the CAMSA skill score (*p* > 0.05) comparing with healthy body mass participants.

The Pearson’s correlation coefficient (see Table 3) indicated a strong relationship (*p* < 0.01) between the CAMSA completion time and three selected agility tests: the IAT (*r* = 0.77), RSS-1MD (*r* =  − 0.76), and RSS-HHD (*r* =  − 0.77). This significant relationship was also found in the CAMSA time score (IAT, *r* =  − 0.79; RSS-1MD, *r* = 0.76; RSS-HHD, *r* = 0.78). In addition, the result also indicated a strong relationship between two versions of RSS (*r* = 0.81, *p* < 0.01), and between the IAT and two versions of RSS (RSS-1MD, *r* =  − 0.79; RSS-HHD, *r* =  − 0.78, *p* < 0.01, respectively).

**Table 3:** Pearson’s correlation matrix of the IAT, RSS-1MD, RSS-HHD, and CAMSA for all study participants (*n* = 149).

**Variables**	**IAT(s)**	**RSS-1MD(steps)**	**RSS-HHD(steps)**	**CAMSA**			
				*Completion time(s)*	*Time score*	*Skill score*	*Total score*
**IAT (s)**	1						
**RSS-1MD (steps)**	−0.79^*^	1					
**RSS-HHD (steps)**	−0.78^*^	0.81^*^	1				
**CAMSA**							
*Completion time (s)*	0.77^*^	−0.76^*^	−0.77^*^	1			
*Time score*	−0.79^*^	0.76^*^	0.78^*^	−0.97^*^	1		
*Skill score*	−0.40^*^	0.48^*^	0.37^*^	−0.49^*^	0.46^*^	1	
*Total score*	−0.76^*^	0.77^*^	0.74^*^	−0.94^*^	0.95^*^	0.72^*^	1

**Notes.**

* Significant level *p* < 0.01.

IAT Illinois Agility Test RSS-1MD Repeated Side Step1 m distance RSS-HHD Repeated Side Stephalf of height distance CAMSA Canadian Agility and Movement Skill Assessment

## Discussion

This study was to evaluate the performance of the CAMSA in Chinese male children and established the validity evidence of the CAMSA timing components. The main finding of this study indicated that older age was contributed to better performances of the RSS-HHD and CAMSA timing components. Better performances of the CAMSA, as in the IAT, RSS-1MD, and RSS-HHD, were associated with having athletic experiences after adjusted sprinting. There was no significant association between body composition and the CAMSA skill score, either being overweight or obese, compared with healthy body mass children. Moreover, the concurrent validity between the CAMSA timing components and the IAT, RSS-1MD, and RSS-HHD were strong, respectively, as we expected.

Consistent with the original Canadian study (Longmuir et al., 2017), our findings illustrated the same evidence of the association between older ages and higher scores of the CAMSA. The inter-rater reliability of the CAMSA skill scores in this study (four examiners) was in line with the prior Canadian study obtained with seven examiners (ICC = 0.66–0.70). However, based on the Canadian criterion (e.g., CAPL-2 edition), 90 (60.4%) of the male children who participated in this study did not meet the recommended levels of physical literacy with a total score categorized as “Beginning and Progressing”, while the rest of participants that did meet this criterion were categorized as “Achieving” (e.g., 22 participants) or “Excelling” (e.g., 37 participants). Even though male children within this exploratory study demonstrated a relatively lower proficiency in the CAMSA, we should note that this finding was referred to the original version of the evaluation norm, which was based on a large cross-sectional study of Canadian children. Regional differences should be considered in the evaluation normalized scoring of the CAMSA when applied in other countries. However, based on the present results, it can be suggested that the CAMSA is a reliable assessment in male Chinese children of different ages, body mass, and athletic experiences. A longitudinal study with more focus on development of fundamental movement skills in Chinese children using CAMSA protocol is therefore suggested.

Although an acceptable correlation was presented between the CAMSA timing components and the IAT, as well as the original version and the new height-based repeated side step we had designed, this does not suggest that the value of the three selected agility tests could be replaced by performing the CAMSA and be simply reflected from the time score of the CAMSA. It is also likely that an individual’s agility might not be fully interpreted by one single agility test considering the complex movement and direction changes required in real-life physical activity and sports. It is possible to hypothesize that better agility needs to be depicted as an ability to be adapted to multiple types of movements. Noted that the agility test generally contains different constructions of movement, distance, direction, route, and tasking process (timing or times), each test should be considered alone as a unique aspect for agility (change of direction in long or short distance, frontal, transverse and sagittal moving direction) (Raya et al., 2013). Although the CAMSA involved different types of movement and direction changing, which is considered as one advantage comparing to traditional agility tests, a note of caution is due here since we might not able to call the CAMSA as “gold standard”, because we selected the timing components of the CAMSA as the indicator of agility, but the whole idea of the CAMSA was to evaluate not only this aspect, but also the dynamic movement skills. However, based on the present study, it can be suggested that the completion time and time score of the CAMSA could be used to evaluate an individual’s agility capacity in comparison with the IAT (with the CAMSA completion time, *r* = 0.77; time score, *r* =  − 0.79), the RSS-1MD (with the CAMSA completion time, *r* =  − 0.76; time score, *r* = 0.76), and the RSS-HHD (with the CAMSA completion time, *r* =  − 0.77; time score, *r* = 0.78). Furthermore, the correlation between this newly designed RSS-HHD and the original RSS-1MD was strong, though, the study of the RSS-1MD was preliminary. There is abundant room for further progress in establishing the validity of RSS-HHD and/or other height-based agility protocol,

One interesting result of this study was that children with athletic experiences had no advantage in the CAMSA skill score, although this effect was slight. In addition, in this study, we found that most of the participants (44 of 46 participants with athletic experiences, 89 of 103 without athletic experiences) could achieve full points for kicking, but demonstrated relatively lower scores in throwing (at the target) and one-foot hopping, which requires higher agility capacity and balance to change direction quickly over short distances (S-type hopping). Within the scoring criterion for “uses overhand throw and hits the target”, participants in this study were typically awarded one point for “transfers weight and rotates body to assist throwing” but were less likely to receive a point for accurately hitting the target. More specifically, only 29 participants (10 with athletic experiences) received the point for accurately hitting the target. It can be argued that these results may be due to low moderate-to-vigorous intensity daily physical activity level (Fan, Chen & Cao, 2017), also the PE class content in China (Ji & Ma, 2019), as the traditional physical education and health course teaching with excessive emphasis on single technology (e.g., standing long jump, ball dribbling, throw a solid ball with both hands as far as they can and no need to aim at a specific target, as these skills will be tested in an entrance exam) might not improve students’ interest in learning, further students’ physical literacy. Moreover, this “floor effect” of the throwing is contrary to a prior Australian study (Lander et al., 2015) which has suggested that the distance of throwing/kicking line is not long enough for students to demonstrate those two skills with a forceful execution. Although we do not fully agree with this idea based on the presented data, we would like to suggest adjusting the difficulty of kicking; one idea is to shorten the “goal area” between two cones instead of making the distance longer, which may also increase the difficulty of throwing.

As required by CAPL, participants should be told that both the completion time and performance of each skill would be scored. It is possible that the examiners may have biased their focus on speed unintentionally by instructing students to “go as fast as you can”.

Although the instruction of “Emphasize that the best score will be retained if they complete the course as fast as they can while doing their best skills” is mentioned in the CAPL, it is possible that participants were had bias on shorter completion time. Children may have realized that they could “sacrifice” the quality of movements to shorten the completion time for a higher total score. The time score lost in “aiming at the target” were more than the point they might earn by more accurately performing the “hit the target”. The previous study also indicated such concern about imbalance between quality of the skills and the time it takes to finish quickly (Lander et al., 2015). This biased strategy might explain the lower proficiency in throwing skill in this study, and might further suggest that the reliability of the CAMSA as an imitation effect and may be indicative of one’s mimicry skills rather than fully understanding the skill technique and criterion (Giblin, Collins & Button, 2014) especially in a short period of time. However, we do not see adding more trials as a better solution, which might make testing less efficient to real-world conditions, and less testing time is one essential advantage of the CAMSA comparing with other FMS assessment. One possible way is to adjust the weight of time and skill score, or to deduct score when participants performed clear bias during testing. However, the suggestion must be interpreted with caution, research is needed to establish the feasibility of this possible modification.

This exploratory study was aimed to report a practical application of the CAMSA in Chinese children for the first time. However, the homogeneity of our participants could be considered a study limitation as only male students were included. Gender differences in movement skills have been illustrated previously (Hardy et al., 2012; Sekulić et al., 2013) that female children are more proficient in performing locomotion skills and stability skills, while male children seem to have an advantage performing object control skills, which may lead to the different CAMSA scores. In future investigations, which take both male and female children into account, it might be possible to build the Chinese version of the CAMSA evaluation norms before large-scale application.

## Conclusions

The data show that, overall, there was positive pattern of older age, having athletic experiences, and being non-obesity in better CAMSA total score, although most of the participant was still at a relatively low level referring to the Canadian criterion. The concurrent validity of the CAMSA completion time and time score were strong in comparison with the IAT, RSS-1MD, and RSS-HHD.

##  Supplemental Information

10.7717/peerj.8784/supp-1 File S1 Raw data

## Additional Information and Declarations

### Data Availability

The following information was supplied regarding data availability: The raw measurements are available in the Supplemental Files.
